# Reducing the lipase LIPE in mutant α-synuclein mice improves Parkinson-like deficits and reveals sex differences in fatty acid metabolism

**DOI:** 10.1016/j.nbd.2024.106593

**Published:** 2024-07-04

**Authors:** M.A. Adom, W.N. Hahn, T.D. McCaffery, T.E. Moors, X. Zhang, P. Svenningsson, D.J. Selkoe, S. Fanning, S. Nuber

**Affiliations:** a Ann Romney Center for Neurologic Diseases, Brigham and Women’s Hospital and Harvard Medical School, Boston, MA 02115, United States of America; b Neuro Svenningsson, Department of Clinical Neuroscience, Karolinska Institutet, 17176 Stockholm, Sweden

**Keywords:** Alpha-synuclein, Hormone-sensitive lipase, Lipe, Parkinson’s disease

## Abstract

Impaired lipid metabolism is a risk factor for Parkinson’s disease (PD) and dementia with Lewy bodies (DLB) and can shift the physiological α-synuclein (αS) tetramer-monomer (T:M) ratio toward aggregation prone monomers. A resultant increase in phospho-serine 129+αS monomers associating with excess mono- and polyunsaturated fatty acids contributes to the αS aggregation. We previously reported that decreasing the release of monounsaturated fatty acids (MUFAs) by reducing or inhibiting the hormone sensitive lipase (LIPE) reversed pathologic αS phosphorylation and improved soluble αS homeostasis in cultured αS triplication PD neurons and reduced DAergic neurodegeneration in a *C.elegans* αS model. However, assessing LIPE as a potential therapeutic target for progressive PD motor phenotypes has not been investigated. 3K αS mice, representing a biochemical and neuropathological amplification of the E46K fPD-causing mutation, have decreased αS T:M ratios, lipidic aggregates, and a L-DOPA responsive PD-like motor syndrome. Here, we reduced LIPE by crossings of 3K mice with LIPE null mice, which attenuated motor deficits in male LIPE^+/−^ knockdown (LKD)-3K mice. Heterozygous LIPE reduction was associated with an improved αS T:M ratio, and dopaminergic neurotransmitter levels and fiber densities. In female 3K-LKD mice, an increase in pS129+ and larger lipid droplets (LDs) likely decreased the benefits seen in males. Reducing LIPE decreased MUFA release from neutral lipid storage, thereby reducing MUFA in phospholipid membranes with which αS interacts. Our study highlights fatty acid turnover as a therapeutic target for Lewy body diseases and support LIPE as a promising target in males. LIPE regulation represents a novel approach to mitigate PD and DLB risk and treat disease.

## 1. Introduction

Familial Parkinson’s disease (fPD) is an autosomal-dominant progressive neurodegenerative disorder. The mutations responsible for PD are N-terminal substitutions in the SNCA gene that leads to disturbed homeostasis of the soluble alpha-synuclein (αS) protein. Neuropathologically, PD is characterized by neuronal loss in several brain regions, most prominently in the midbrain and cortex, as well as deposition of cytoplasmic αS-containing aggregates, called Lewy-bodies (LBs). LB in human (hu) PD brain can also contain lipid droplets and membrane fragments. Our and other labs have shown that the physiological state of αS includes the formation of aggregate-resistant tetramers and these can be decreased due to excessive monomer binding to lipid membranes (Bartels et al., 2011; Dettmer et al., 2013; Dettmer et al., 2015a; Dettmer et al., 2015b; Wang et al., 2011; Westphal and Chandra, 2013; Burre et al., 2014; Trexler and Rhoades, 2012; Kim et al., 2018; Gurry et al., 2013). The relevance of tetramer:monomer (T:M) balance to disease was established when fPD-causing αS missense mutations were all found to decrease the physiological T:M ratio in neurons, thereby increasing the levels of aggregation-prone monomers (Dettmer et al., 2015a; Tripathi et al., 2022). In the case of the E46K fPD mutation, adding analogous *E*-to-K mutations into the adjacent KTKEGV repeat motifs (at residues 35 and 61, thus “3K”), further decreased tetramers and caused neuropathology and progressive motor deficits, closely resembling PD^12^. 3K αS mutant mice accumulate an excess of triacylglycerols (TAGs), and LDs (a major storage pool for TAGs) in vulnerable (DAergic and cortical) brain neurons (Nuber et al., 2021; Nuber et al., 2022), resulting in impaired lipophagy that can be rescued by transducing the lysosomal enzyme glucocerebrosidase (GCase) (Glajch et al., 2021). Our studies further showed that overexpressing wild-type GBA1 (coding for GCase) in the 3K αS mutant mice increased soluble, physiological tetramers, associated with fewer lipid-membrane-rich aggregates (Glajch et al., 2021), similar to what has been observed in human PD neurons (Kim et al., 2018).

Thus, one approach to alleviate αS toxicity is targeting activities of proteins involved in lipid homeostasis, including enzymes. Research from several labs including ours showed that inhibiting the synthetic enzyme for MUFAs, stearoyl-CoA desaturase (SCD), in PD patient-derived neurons ameliorated the neurotoxicity (Nuber et al., 2021; Nuber et al., 2022; Fanning et al., 2019; Vincent et al., 2018; Imberdis et al., 2019), and improved numerous PD-like motor phenotypes in the 3K model but also in mildly impaired WT αS tg mice (Nuber et al., 2021; Nuber et al., 2022). The SCD enzyme catalyzes the rate-limiting in the formation of palmitoleic acid, C16:1 and oleic acid, C18:1, which can increase membrane-fluidity and may undergo trans-esterification into TAGs. The inhibition of SCD improved the αS T:M ratio and decreased abnormal membrane-associated αS and lysosomal and lipid droplet-rich aggregates and improved lysosomal enzyme maturation - such as glucocerebrosidase and cathepsins- thereby removing excess MUFAs and TAGs in vivo (Nuber et al., 2021; Nuber et al., 2022).

On the other hand, unsaturated fatty acids can also be generated by lipase degradation pathways. Here, we test LIPE (also named hormone-sensitive lipase, HSL), the rate-limiting enzyme in this process, as a candidate PD/DLB therapeutic target in vivo. Our previous study in PD-patient derived neurons with αS gene dosage or missense mutations showed that knocking down or pharmacologically reducing LIPE decreased MUFAs in phospholipid membranes and simultaneously restored the soluble αS T:M homeostasis (Fanning et al., 2022). In order to dissect lipid metabolism by reduction of LIPE in vivo, LIPE ^−/−^ (also named HSL^−/−^) mice were created (Li et al., 2002; Haemmerle et al., 2002) and a reduction in TAGs and other FAs were observed in the fasted state with interim (heterozygous) levels in the LIPE^+/−^ (LKD) mice. A complication of LIPE is a sex-linked reduction in expression and enzymatic activity in cycling female mice (Stelmanska et al., 2015).

We therefore chose crossings between 3K and LKD (^+/−^) mice to only partially reduce LIPE enzymatic activity in the 3K αS mouse brain (offspring termed 3K-LKD). We found that knocking down one allele of LIPE improved physiological αS T:M homeostasis in mouse brain and reduced pS129+ aggregates and this was associated with increased compensatory (dopaminergic and serotoninergic) neurotransmitter turnover and TH+ fiber densities, and improved motor skill learning and gait regularity in male 3K-LKD mice. We identified a distinct sex difference, with female 3K-LKD mice accumulating pS129 + αS and larger sized lipid droplets (LDs) that may hinder LIPE benefits observed in male mice. Lipid profiling of 3K-LKD showed a reduction of certain MUFAs, belonging to the class of phosphotidylethanolamine-ethers, in male 3K-LKD but much less in female 3K-LKD, that can enhance αS aggregation and neuronal vulnerability.

## 2. Results

### 2.1. Heterozygous deletion of LIPE more pronouncd in females than in male 3K αS mutant mice

**Fig. 1.**
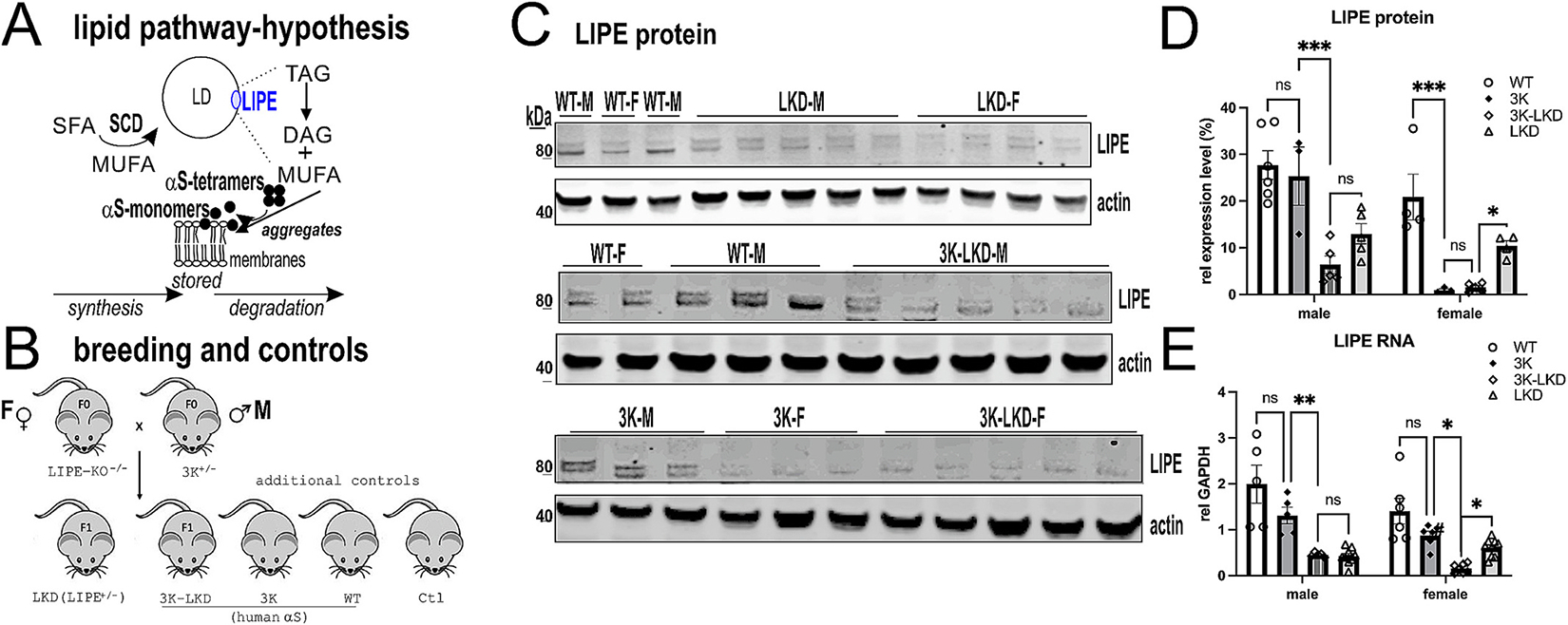
Reducing LIPE in 3K mice. (**A**) Neutral lipid synthesis and degradation pathway hypothesis: A disequilibrium of SFA and MUFA in PD-type mice leads to excess build-up of TAGs (stored in LDs) and changes in saturation of phospholipid membranes, disturbing αS membrane association and thereby the αS T:M homeostasis, and this can be improved by SCD enzymatic inhibition. Inhibiting LIPE reduces multiple FA degradations and thereby excess turnover of TAGs generating MUFAs that can promote lipidic αS+ aggregates. (**B**) Mouse schematic depicting breeding strategy. LIPE null (^−/−^) (also known as HSL, hormone sensitive lipase) were crossed with 3K αS mutant mice, producing heterozygous 3K-LKD and LKD mice. Sex and age-matched controls for human αS homeostasis (hu WT αS and 3K αS) and non-transgenic C57Bl6 mice (Ctr) were added to the study. (**C**) WB shows the predicted short isoforms (80 and 83KDa) of the LIPE protein in WT, 3K and expected reduced level in LKD, 3K-LKD mouse brain cortex. (**D**) Quantifies LIPE protein in mouse brain cortex. (**E**) LIPE mRNA expression level in mouse cortex relative to GAPDH. LKD reduced LIPE RNA vs. WT and 3K, and this was further reduced in female 3K-LKD vs male 3K-LKD. Data are mean ± SEM. 2-way ANOVA, post Tukey. *p < 0.05, **p < 0.01, ***p < 0.001, ns non-significant. Ctl, control (non-transgenic); DAG, diacylglycerides, TAG, triacylglycerides; F, female; LKD, LIPE knockdown; M, male; MUFA, monounsaturated fatty acid; SCD, stearoyl-CoA desaturase, SFA, saturated fatty acid; WT, wildtype;

Our working hypothesis is that both FA synthesis or degradation pathways can lead to an increase in MUFAs, promoting a shift from αS tetramers to excessive monomers, interacting with membrane lipids and their consecutive aggregation (Fig. 1A). We used heterozygous LIPE knockdown (LKD) mice to evaluate whether a reduction in LIPE level has beneficial effects on synucleinopathy and motor phenotypes of 3K αS mutant mouse. For this purpose, we crossed homozygous female LKO (^−/−^) with heterozygous male 3K mice, generating 3K-LKD mice and LKD control mice. Additionally, LKD mice were bred with C57Bl6 control (Ctr) mice, producing both Ctr and LKD F1 mice. Expression- and age-matched 3K αS mutant mice as well as WT (human wildtype) αS mice were used as controls for analyzing changes in hu αS tetramerization and solubility vs. 3K-LKD mice (Fig. 1B).

We confirmed the deletion of 1 LIPE allele at the protein level by western blot analyses and the RNA level by RT-qPCR prepared from cortices of 6 months old mice. Since LIPE expression was reported to be regulated by sex hormones in female rodents (Stelmanska et al., 2015; Szafran and Smielak-Korombel, 1998; Samra et al., 1998) we assessed brain expression level of male and female LKD and 3K-LKD mice vs. WT and 3K mice (Fig. 1C–E). The LIPE protein has several isoforms, which are generated by use of alternative translational start codons. The short isoforms (80–90 kDa) are the most commonly detected isoforms (Kraemer and Shen, 2002; Manna et al., 2013; Yuksel et al., 2023), and these were accordingly detected in cortical protein extracts of WT and 3K mice, with the expected lower amounts in the LKD and 3K-LKD mice. Smaller (<80 kDa), truncated isoforms were not included in the quantification since these were reported being inactive (Althaher, 2022) (Fig. 1C,D). Interestingly, both female 3K-LKD and 3K showed a pronounced reduction in the (sum of the) 80–90 kDa isoforms vs. male mice and a two-way ANOVA revealed significantly reduced LIPE protein by genotype (F_3,27_ = 18.57, *p* < 0.001) (LKD vs. WT, ~ 50%) and sex (F_1,27_ = 21.23, *p* < 0.001) (Fig. 1D). RT-qPCR of the mRNA derived from brain cortex of these mice substantiated reduced expression in LKD and this was further reduced in female 3K-LKD vs. male 3K-LKD (Fig. 1E). To test two major lipid groups potentially affected by LIPE, we compared brain TAGs and diacylglycerols (DAG) in 6 months old LKD vs. control brain (Suppl Fig. 1). While lipid profiling of the cerebral cortex only showed a trend (*p* = 0.1) for increased TAGs in LKD vs. Ctl (Suppl Fig. 1A), an expected significant increase was detected in the balance between TAGs with DAGs, either when plotting the ratio of TAGs to total DAGs (Suppl Fig. 1B, *p* < 0.05) or to the sum of total TAGs + DAGs (Suppl. Fig. 1C, *p* < 0.05). The balance between TAGs and DAGs was not influenced by sex in LKD mice (Suppl Fig. 1D). These findings suggest that heterozygous crossings between LKD and 3K led to an expected decrease in LIPE RNA and protein, however, female sex in 3K mice further reduced the LIPE expression level.

### 2.2. LIPE reduction improves gait pattern and motor skill learning in male 3K αS mutant mice

**Fig. 2.**
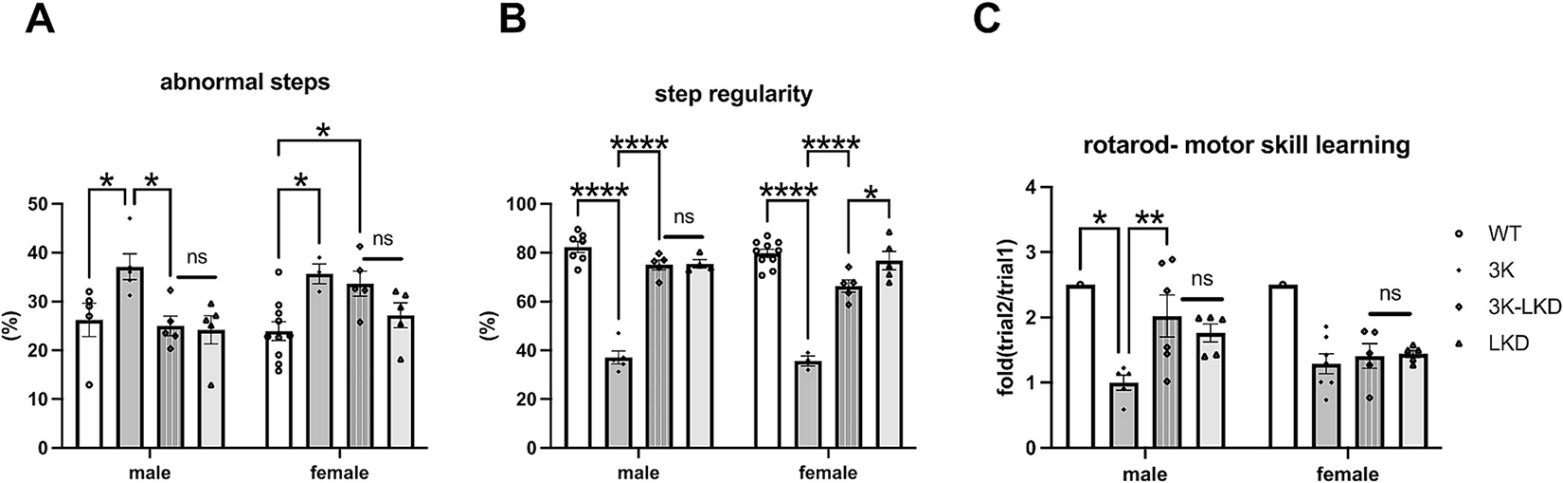
LIPE genetic reduction (LKD) improves the gait impairment, motor skill learning in male 3K mice. (**A**) Automated gait scans of mice paw pattern on a horizontal treadmill (Cleversys) record to quantify step abnormalities and (**B**) step regularity. (**C**) Graph quantifies balancing skill learning on a 4–40 rpm accelerating rotarod. Data are mean ± SEM. *p < 0.05, **p < 0.01, ****p < 0.0001, ns, non-significant. Two-way ANOVA, post Tukey.

We previously showed 3K gait abnormalities associated with impaired motor skill learning that can be attributed to plasticity of cortical, DAergic and other neurons, that improved by SCD inhibition in 3K mice (Nuber et al., 2021; Nuber et al., 2022). Thus, we placed 3K-LKD mice on treadmills to evaluate finer gait patterns and motor skill learning (Fig. 2). Photographic analysis of the gait (GaitScan, Cleversys) showed that reducing LIPE significantly decreased the number of abnormal steps in male 3K-LKD vs. 3K (2-way ANOVA, *p* < 0.05) (Fig. 2A), with no change detected between female 3K-LKD and 3K mice. The gait regularity index, a fractional measure of inter-paw coordination of normal steps relative to total number of paw placements, substantiated improved regularity of gait in male 3K-LKD mice (*p* < 0.0001) and in female 3K-LKD vs. 3K mice (Fig. 2B). To further analyze whether the fine gait changes impacted motor skill learning, these mice were subjected to rotarod testing. Reducing LIPE significantly improved motor skill learning in male 3K-LKD vs. 3K (*p* < 0.01), but these were non-significant (ns) in female mice (Fig. 2C). This suggests LIPE reduction partially improved gait regularity in male and female 3K mice and this was associated with an increase in motor skill learning in male mice.

### 2.3. LIPE reduction improves hu αS solubility in both sexes but reduces abnormal pS129+ only in 3K males

**Fig. 3.**
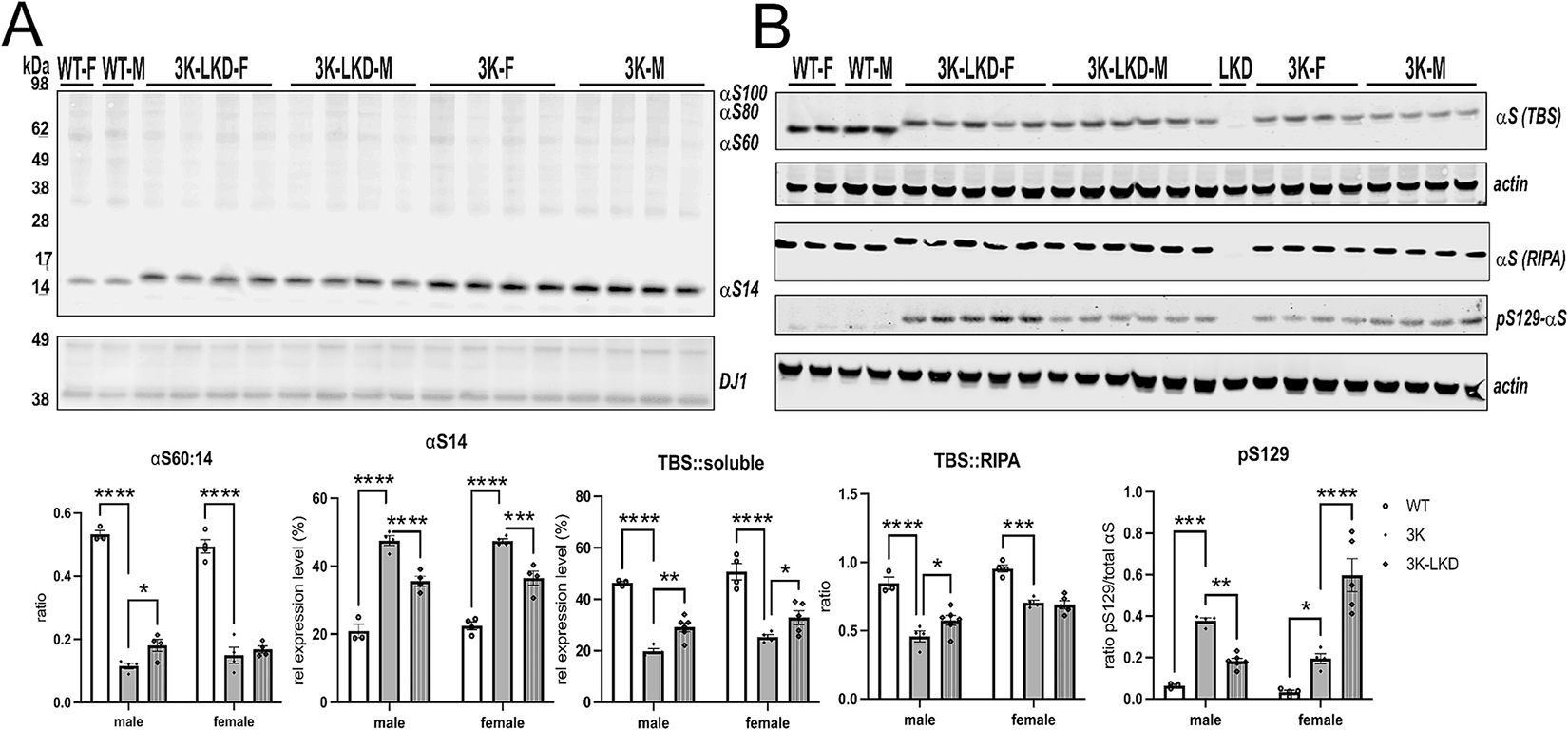
LIPE genetic reduction improves the T:M αS ratio and reduces insoluble pS129 αS in male 3K mice. (**A**) Intact-cell crosslinking of αS using the cell-penetrant cross-linker DSG in cortical brain bits lysed in PBS/ 1% Triton-X100. Below quantification of the rel. Tetramer (αS 60 kDa) to monomer (αS 14 kDa) ratio and monomer level of the corresponding WB. DJ1 serves as a loading and cross-linking control. Quantification reveals the significant decrease in excess monomers by 3K and increased αS T:M in male 3K-LKD vs. 3K (**B**) Representative WBs (non-crosslinked) of sequentially extracted TBS-soluble (cytosolic), RIPA (insoluble) extracts of cortical brain bits. A LKD control (one lane) was loaded between 3K-LKDM and 3K-F. Actin serves as loading control. Data are mean ± SEM. Two-way ANOVA, post Tukey. *p < 0.05, **p < 0.01, ***p < 0.001, ****p < 0.0001.

Our previous studies showed gait regularity correlated with a normalization of the physiological αS T:M ratio in 3K mouse brain (Nuber et al., 2021; Glajch et al., 2021; Rajsombath et al., 2019). We described a method to trap the cell-lysis-sensitive tetramers by intact-cell crosslinking of fresh, minced brain tissue bits with the cell-penetrant crosslinker DSG (Dettmer et al., 2013; Dettmer et al., 2015a). Using this intact-cell crosslinking method we tested whether reducing LIPE altered the human αS tetramers (designated αS60) and related multimers (αS80 and αS100) as well as the monomers (αS14). Similar to our cell culture studies (Fanning et al., 2022), the major effect of LIPE was a significant reduction of excess αS monomers both in male and female 3K-LKD (*p* <0.001) and this still significantly raised the αS T:M ratio in male 3K-LKD vs 3K mice (*p* < 0.05) (Fig. 3A).

Sequential extractions of cerebral cortex (without crosslinking) and western blotting revealed a shift to buffer-soluble (cytosolic) αS in both male and female 3K-LKD vs. 3K mice (*p* < 0.01), and the overall TBS-soluble/RIPA-insoluble ratio increased in male 3K-LKD vs LKD mice (*p* < 0.05) (Fig. 3B). To ensure solubility was not affected by direct effects of female sex, we evaluated male and female LKD mice and found no significance between sex and when compared to WT control mice (Suppl Fig. S2).

Probing for pS129, a marker for aggregated αS in hu PD and DLB, revealed lower signals in male 3K-LKD vs 3K mice. However, an unexpected increase in phosphorylated S129 monomer signal was found for female 3K-LKD vs. 3K mice (Fig. 3B, 2-way ANOVA, *interaction* F_2,20_ = 32.05, *p* < 0.0001). No pS129 specific signals were detected in LKD mice (see representative lane “LKD”, Fig. 3B).

### 2.4. LIPE reduction improved TH+ fiber densities and neurotransmitter turnover in male 3K αS mutant mice

**Fig. 4.**
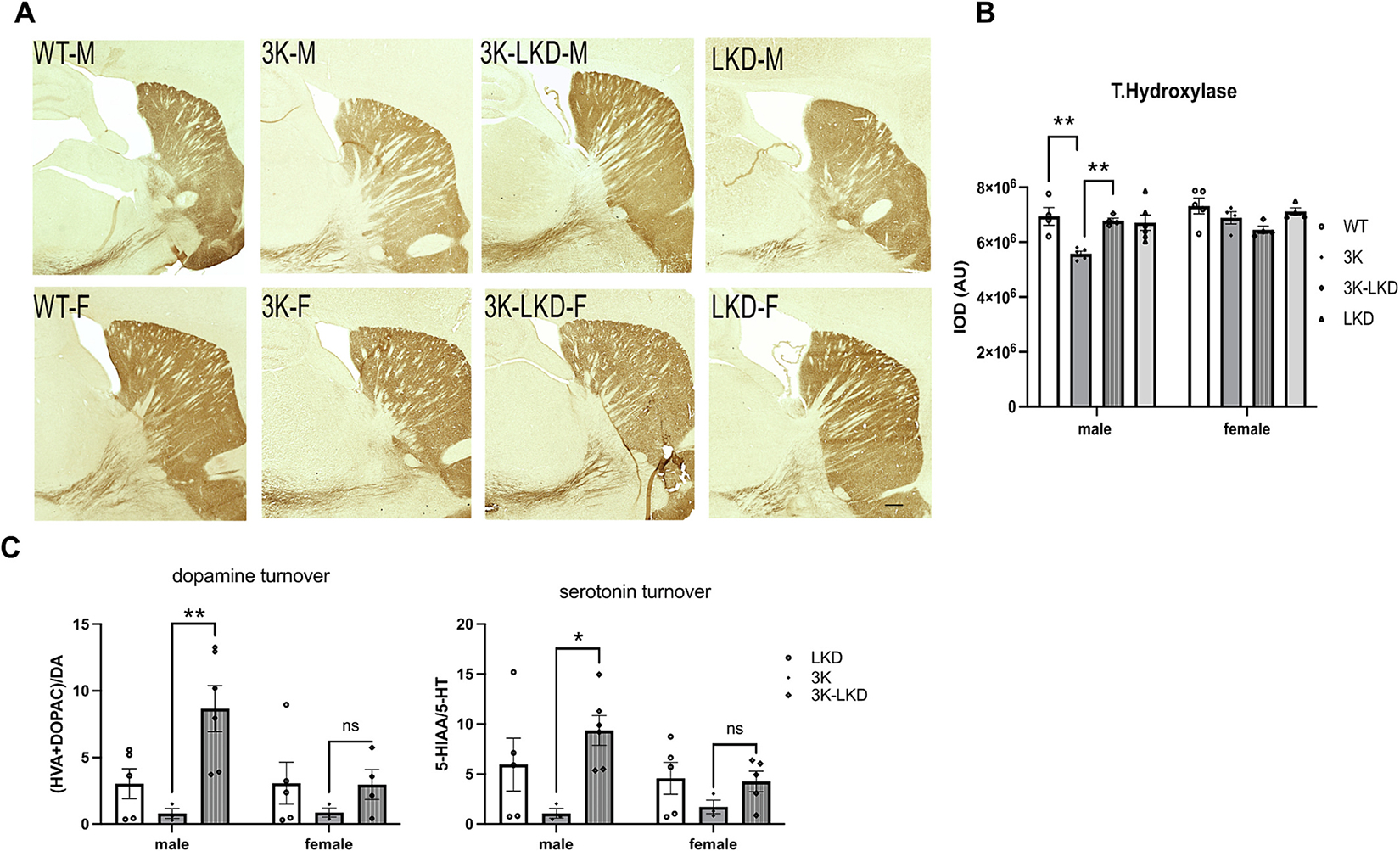
TH+ striatal fibers and turnover of striatal dopamine and serotonin by LIPE reduction in male and female 3K. (**A**) Representative images of tyrosine hydroxylase (TH) positive nerve terminals and fibers of male and female WT, 3K, 3K-LKD and LKD mice. Scale bar, 600 μm. (**B**) Relative TH optical density (total of 12 sections; *n* = 3–4 mice each cohort) was analyzed in the dorsal striatum. (**C**) HPLC assay of striatal DA turnover (ratio of metabolites, DOPAC + HVA/DA) and serotonin turnover (ratio of (5-HIAA)/5-HT) in mice. Data are mean ± SEM. 2-way ANOVA, post Tukey. *p < 0.05, **p < 0.01. DA, dopamine: DOPAC, 3,4-Dihydroxyphenylacetic acid; HVA, homovanillic acid; 5-HIAA, 5-Hydroxyindoleacetic acid; 5-HT, 5-hydroxytryptamine.

Given the improvement in the physiological αS T:M ratio and gait parameters in 3K-LKD, we quantified tyrosine hydroxylase immune-positive (TH+) axons and their terminals. We analyzed the dorsal striatum, which is rich in projections from the DAergic neurons of the substantia nigra pars compacta (SNpc). As previously shown (Rajsombath et al., 2019), male (but not female) 3K mice have less striatal TH+ vs. WT mice (Fig. 4A). Reducing LIPE led to an ~20% increase of TH+ fiber density in male 3K-LKD vs. 3K but there were no significant changes between female mice (Fig. 4B)

The increase in striatal nerve fibers and motor skill learning suggests improved synaptic plasticity. Therefore, we assessed dopamine (DA) and serotonin metabolism by HPLC. An increase in DA and serotonin metabolism is a known compensatory mechanism of functional synapses early in PD (Wile et al., 2017; Lee et al., 2000). Thus, we measured DA and serotonin against their metabolites 3,4-Dihdroxyphenylacetic acid (DOPAC) and homovanillic acid (HVA) or 5-HIAA, respectively. Quantification of the HPLC measurements showed a significant increase in neurotransmitter turnover in male 3K-LKD vs. LKD and a trend for female 3K-LKD mice (Fig. 4C). A 2-way ANOVA showed significant genotype effects (F_2,21_ = 4.268, *p* = 0.02) but no significant interaction or differences by sex. This finding is in accord to a previous finding, that reducing LIPE protected against DAergic degeneration in a PD worm model (Fanning et al., 2022).

### 2.5. LIPE reduction reduces pS129+ inclusions, LD size and distribution in male 3K αS mutant mice

**Fig. 5.**
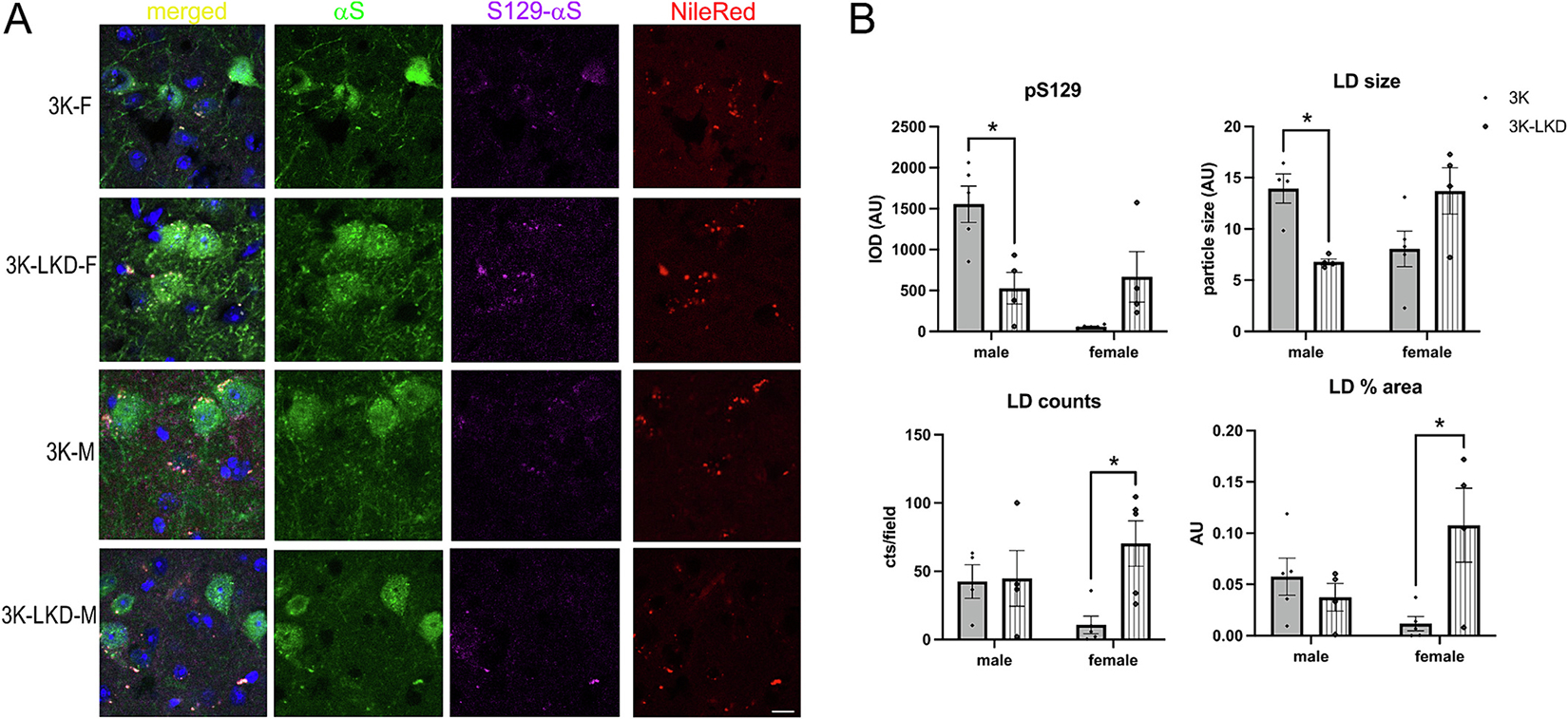
LIPE reduction decreases LDs in neurons with pS129α_+_ in male 3K but their excess is detected in female 3K mice. (**A**) Confocal microscopy of cortical sections quadruple labeled with Nile Red (red), pS129 (magenta), αS (green) and DAPI (blue). (**B**) Quantification of pS129+ puncta showed a decrease in male 3K-LKD consistent with a decrease in LD size and distribution. Note fewer αS + fiber varicosities in male 3K-LKD vs. 3K. Two-way ANOVA, post Tukey. *p < 0.05. Scale bar, 25 μm. (For interpretation of the references to colour in this figure legend, the reader is referred to the web version of this article.)

Abnormal, membrane-lipid rich aggregates are found in human PD brain (Shahmoradian et al., 2019), in PD-GBA patient derived neurons (Schondorf et al., 2014) and in PD fly models (Girard et al., 2020). We consistently observed pS129+ multilaminar membranes and some LB-like aggregates in PD-type 3K αS mice (Nuber et al., 2018). Nile Red is a histochemical marker for neutral lipids and LDs. We previously showed that SCD inhibition (Nuber et al., 2022) or increasing enzymatic function of GCase (Glajch et al., 2021) reduced the lipid-rich (Nile Red+ and pS129+) deposits in cortical and midbrain neurons in 3K mice. Therefore, we next quantified Nile Red+ puncta in male and female 3K-LKD vs. 3K mice (Fig. 5A). Reducing LIPE significantly reduced pS129+ puncta and their colocalization with sizeable LDs in male 3K-LKD vs. 3K, while LD markers (size and distribution) in pS129+ neurons were increased in female 3K-LKD vs 3K (2-way ANOVA, *p* < 0.05) (Fig. 5B). This suggests a sex dimorphism in LDs associating with pS129+ in 3K-LKD mice.

### 2.6. LIPE reduction modulates MUFA incorporation into phospholipid membranes in 3K mouse brain

**Fig. 6.**
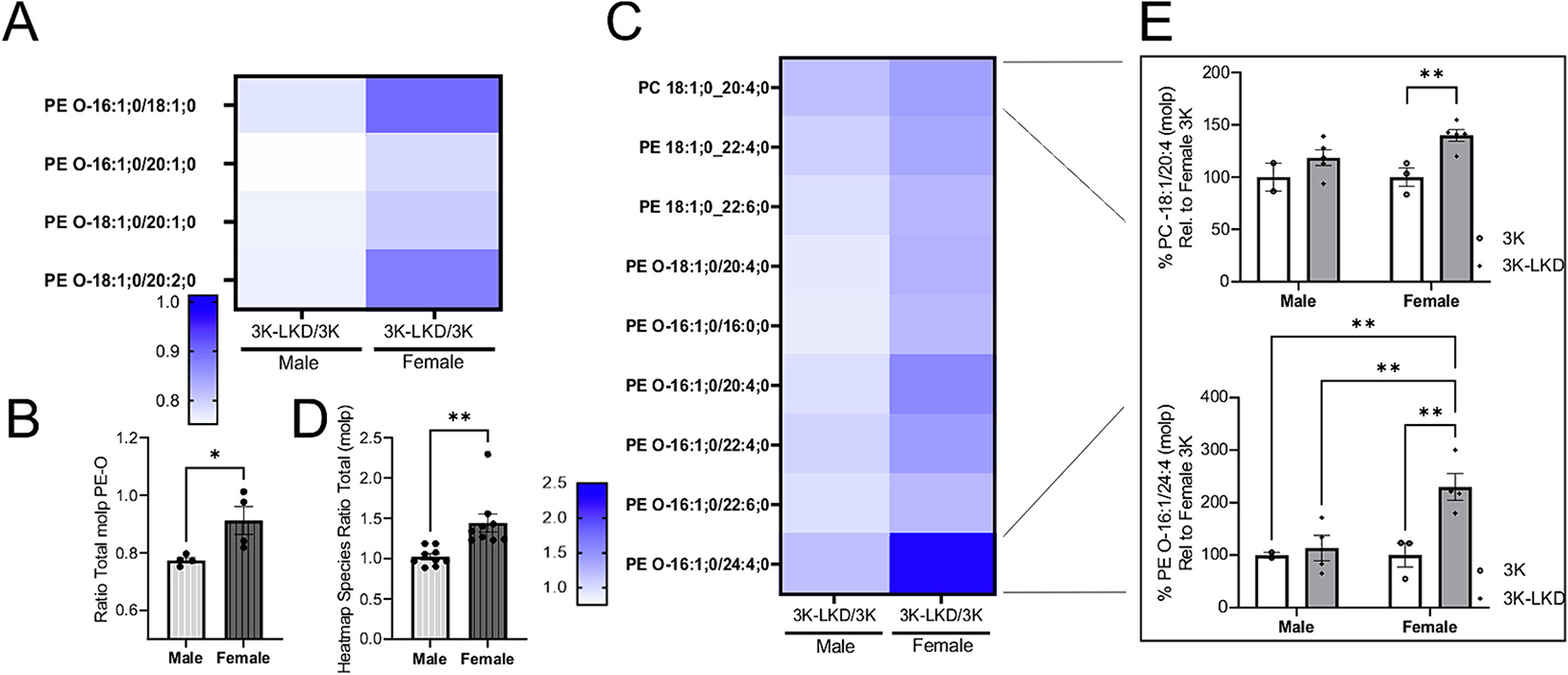
LIPE Reduction re-balances MUFA-containing phospholipids. (**A**) PE-O containing C16:1 or C18:1 decreased in male 3K-LKD/3K mice. Ratios for each phospholipid species were calculated for 3K-LKD mice vs 3K (molp) for males and females. Those phospholipids with >20% decrease in males (that were not also decreased in females) were represented in the heatmap. Graph in **(B)** below quantifies the ratio of total PE-O between male and female mice (unpaired two-tailed *t*Test, *p < 0.05). (**C**) PE-O containing C16:1 or C18:1 increased in female 3K-LKD/3K mice. Ratios for each phospholipid species were calculated for 3K-LKD mice vs 3K (molp values) for males and females and are represented in the heatmap. (**D**) Graph quantifies total phospholipids with >20% increase in females and that were not also increased to the same degree males. (**E**) Two examples of female only 3K-LKD vs. 3K differences in UFA for PC and PE-O lipid classes are C16:1 or C18:1 containing lipids, and PE-O 16:1/24:4 was significantly more in female 3K-LKD vs 3K or vs. male 3K or 3K-LKD. Data are mean ± SEM. Two-way ANOVA post-hoc Tukey. *p < 0.05. ** *p* < 0.01. Graph Pad Prism Heatmap Scale bar.

We previously showed a significant accumulation of unsaturated FAs containing TAGs including increased TAG 50:1 and TAG52:1 in male 3K vs WT mice. These were significantly reduced by inhibiting SCD, the key enzyme of MUFA generation (Nuber et al., 2021). We identified LIPE reduction as a candidate therapeutic approach premised on preventing the aberrant increase in MUFAs, stored in the neutral lipid (e.g. TAG) form being cleaved from neutral lipids and incorporated into phospholipid membranes (Fanning et al., 2022). LIPE reduction maintains a portion of the MUFAs in neutral form, resulting in reduced PD-associated phenotypes. To determine the phospholipids altered upon LIPE reduction that associated with improved PD phenotypes, we assayed the mouse brain lipidome focused on MUFAs in the 6 mos male and female 3K, 3K-LKD mice (Fig. 6). We first focused on the set of C16:1 or C18:1 containing phospholipids with an average decrease of >20% in 3K-LKD vs 3K male mice, associating with improved phenotypes in male 3K-LKD mice. Interestingly, all phospholipids in this group belonged to the phosphotidylethanolamine-ether (PE-O) class of lipids (Fig. 6A). However, these lipids were not decreased by 20% in the female 3K-LKD vs 3K comparison (Fig. 6A, graph in B). We then analyzed the phospholipids containing C16:1 or C18:1 that had an >20% increase in 3K-LKD vs 3K females to establish the alterations to membranes correlated with female mice in which most of the phenotypes were not rescued to the extent of that observed in male 3K-LKD (Fig. 6C). Importantly, this group was also enriched for PE-O lipids and contained phosphotidylethanolamine (PE) and phosphotidylcholine (PC) lipids. Those increased by >20% in 3K-LKD vs 3K females and were not increased to the same extend (by >20%) in the male comparison (Fig. 6C, graph in D). We found a significant increase of PC 18:1_20:4 in female 3K-LKD vs 3K but not in male 3K-LKD vs 3K (Fig. 6E). In addition, the PE-O species PE-O 16:1_24:4 was significantly increased in females vs males (Fig. 6E). These results suggest PE-O MUFA membrane composition may help determine beneficial effects of LIPE on hu αS membrane interactions impacting αS disease biochemical readouts and PD phenotypes that we detect in male 3K-LKD mice in vivo.

## 3. Discussion

Our results show that αS solubility, αS T:M disequilibrium and motor abnormalities in male 3K αS PD mouse model mice can all be improved by decreasing the major lipid degrading enzyme, LIPE. Using the 3K αS mouse, which was designed to reproduce certain classical PD phenotypes based on the biochemical instability of physiological αS tetramers, we determined that brain neurons develop lipid-rich αS inclusions in the core biological processes implicated both in familial and sporadic human PD: lipid metabolism, DAergic fiber integrity and motor function (Nuber et al., 2018). Because the αS T:M ratio is increasingly supported as a relevant measure of αS pathology and mediated by fatty acyl membrane compositions (Kim et al., 2018; Nuber et al., 2018; Wooddell et al., 2017; Fanning et al., 2018), we measured the effects of LIPE enzyme downregulation on this αS homeostasis and motor function. LIPE KD significantly decreased the excess monomers and increased the αS T:M ratio in male 3K-LKD. We observed a similar trend in αS T:M homeostasis in female 3K-LKD but this did not reach statistical significance.

LIPE reduction did not rescue most phenotypes studied in symptomatic female 3K mice, which displayed increased pS129+ monomers and LD sizes. Specifically, we observed a sex dimorphism in PE-O – with the decreased PE-O containing MUFA species in male 3K-LKD mice associating with a rescue of PD phenotypes in vivo – while increased MUFA PE-O species were found in phenotypically abnormal female 3K-LKD. PE-O is a significant contributor to neural membrane structures and properties impact vesicle interactions, trafficking, and signaling and their abundance can result in ferroptosis. The finding of decreased C16:1 and C18:1 containing PE-O species upon genetic LIPE reduction in vivo is in-keeping with our findings of decreased C16:1 and C18:1 containing PE-O species upon LIPE reduction using two diverse LIPE-specific inhibitors (13 g and BAY) in patient-derived iPSC neurons in vitro (Fanning et al., 2022). Collectively these data support the hypothesis that PE-O membrane composition impacts PD-relevant phenotypes and a mechanistic role of LIPE reduction in re-balancing PE-O homeostasis in cytoplasmic membranes.

DAergic and other neurons contribute to the rotarod deficit in the 3K mouse model (Nuber et al., 2018), which is according to studies showing rotarod motor skill learning require an intact circuit between cortex, cerebellum and basal ganglia (Tian and Chen, 2021). Thus, the cortical neuropathology, including hyperphosphorylated αS, may contribute to the motor deficits observed in female 3K-LKD. The dimorphism by sex in 3K mice may correlate with the amount of LIPE, since heterozygous crossings of female 3K and LKD mice reduced LIPE by much more than 50% (~80–90%). Interestingly, previous reports showed hormonal (i.e., estrogen, insulin) or transcriptional factors (i.e., polymorphisms) can repress approximately 40% of LIPE expression (Stelmanska et al., 2015; Szafran and Smielak-Korombel, 1998; Talmud et al., 2005; Talmud et al., 1998), however, this can be an intermittent in these cycling females (Samra et al., 1998). Reducing LIPE by not >50% in male 3K-LKD decreased pS129+ and LD sizes and improved inhibitory DA and 5-HT neurotransmitter turnover. Thus, an imbalance between inhibitory and excitatory neurotransmission may explain the raise in pS129 (Ramalingam et al., 2023) in female 3K-LKD, decreasing tetramer-stabilization and the behavioral benefits seen in male 3K-LKD.

Women with PD have overall milder symptoms and later age of onset, thus an moderate intermittent reduction (~40%) of LIPE in female hu PD could be beneficial during the aging process. There are other established epidemiological and clinical distinctions between men and women with PD and the molecular basis of sex differences remain largely unexplored. Thus, understanding the mechanisms that reduced LIPE expression lifelong in female mice may help to further dissect the relevant sex difference in these PD-type mice (Rajsombath et al., 2019; Moors et al., 2023).

In previous studies we used SCD inhibitors and overexpression of wildtype GCase to reduce the excess MUFAs and LDs (storing trans-esterified MUFAs) (Nuber et al., 2021; Nuber et al., 2022; Glajch et al., 2021). In the current study we used a genetic approach to produce a stable decrease in neutral lipids degradation pathway – evidenced by the disbalance between TAGs and DAGs – as a controlled approach to analyze modulation of MUFA release and membrane phospholipid incorporation.

In summary, decreasing LIPE in progressive PD-type 3K mutant αS male mice reduced αS lipid-membrane association, improved the soluble αS T:M homeostasis, compensatory neurotransmitter turnover, nerve fiber integrity and of relevance, motor skill learning and gait regularity. It will be important to examine pharmacological inhibition of LIPE using a moderate dose to reduce 10–20% of LIPE in both female and male 3K and other PD-relevant mouse models to continue evaluating LIPE in preclinical models as a candidate therapeutic target for PD.

## 4. Methods

### 4.1. Mouse models

All procedures on mice were approved by the IACUC and are reported following the guidelines. Mice were housed on a 12-h light-dark cycle with lights on at 7:00, room temperature at 21–23C and humidity at 55–60%, and had access to regular chow and water ad libitum. 3K and WT mice are JAX cryosperm preserved (3K #414469, WT #414486), HSL-knockout (HSL^−/−;^; designated LIPE-Knockout) mice were generated by targeted disruption of the HSL gene (LIPE) as described elsewhere, purchased by the Jackson Laboratory (JAX mice #019004), and backcrossed to C57Bl6 to generated heterozygous LIPE-knockdown (LKD).

### 4.2. Intact-cell crosslinking of brain tissue

Dissected brain regions were gently minced into small bits with a razor blade, and the brain bits were washed free of released cytosol and re-suspended in PBS with EDTA-free Complete protease inhibitors (Roche Applied Science). Intact-cell crosslinking was then conducted on the washed brain bits as previously described (Dettmer et al., 2013) with minor modifications. Briefly, the cell-permeable crosslinker DSG was prepared at 1 mM final concentration in DMSO immediately before use. Samples were incubated with crosslinker for 40 min at 37 °C with rotation. The reaction was quenched by adding Tris, pH 7.6, at 100 mM final concentration and incubated for 10 min at RT. After quenching and aspiration of the supernatant, proteins in intact tissue were extracted directly in TBS/1% Triton X-100 (below).

### 4.3. Sequential tissue extractions

The regional expression pattern of αS was initially examined at age 6 mo. Mice were anesthetized, decapitated and the brains dissected on a chilled stage. Sequential extractions were performed as described (Nuber et al., 2013). Briefly, tissues were homogenized in 2.5 volumes of TBS+ [50 mM Tris-HCl, pH 7.4, 175 mM NaCl; 5 mM EDTA; protease inhibitor cocktail (Calbiochem, CA)] and spun for 30 min at 120,000 *g*. The pellet was subsequently extracted in TBS+ containing 1% Triton X-100, then in TBS+ containing 1 M sucrose, The TX-insoluble pellet was then extracted in RIPA buffer (TBS+, 1% NP-40, 0.5% sodium deoxycholate, 0.1% sodium dodecyl sulphate), with each extraction step followed by ultracentrifugation for 30 min at 120,000 *g*.

### 4.4. Western blot analyses

For western blotting, 10–25 μg total protein of sequential extracts of dissected mouse brain regions were electroblotted onto nitrocellulose membranes (Millipore, Bedford, MA). For improved immunodetection of αS (monomers of which are prone to washing off filters (Newman et al., 2013; Lee and Kamitani, 2011)), the membranes were fixed in 0.4% paraformaldehyde (PFA) for 20 min. After washing in phosphate-buffered saline (PBS), membranes were blocked for 1 h at RT in PBST (phosphate-buffered saline with 0.2% Tween-20) containing 5% bovine serum albumin (BSA). Blots were then incubated with human-specific αS antibody (ab) (15G7, Enzo; 1:500; 4B12, 1:2000; Sigma), or ab against phosphorylated (ser129) αS (51,253; Abcam; 1:5000) of LIPE (4107S; Cell Signaling; 1:1000) in PBST containing 5% BSA overnight. After washing with PBST, membranes were probed with appropriate secondary antibodies (1:5000, American Qualex, CA), visualized with enhanced chemiluminescence (ECL, PerkinElmer, Boston, MA), and analyzed with the VersaDoc gel imaging system. Proteins were normalized to β-actin (A5441, Sigma; 1:3000) or GAPDH () used as a loading control. Quantification of signal intensities was performed as described (Nuber et al., 2008).

### 4.5. High pressure liquid chromatography

HPLC was conducted as previously described (Sousa et al., 2021). To estimate striatal monoamine levels the mice were deeply anesthetized by CO_2_, quickly decapitated, and the striata dissected on ice, homogenized in 0.5 M perchloric acid, centrifuged, filtered, and stored at −80 °C until analysis for monoamine content. Samples containing 500 pg dihydroxybenzylamine as an internal standard were analyzed by HPLC with electrochemical detection. The analysis was performed on HPLC-ECD system (Dionex Ultimate 3000, ThermoFisher Scientific, Waltham, MA, USA). The separation was performed on a C18 reversed-phase column at 30 °C. The mobile phase (75 mM monobasic sodium phosphate, 1.7 mM OSA, 100 μL/L TEA, 25 μM EDTA, and 10% acetonitrile (*v/v*), pH 3.0), was pumped at a flow rate of 0.4 mL/min. The first and second analytical cells were set to −100 mV and + 300 mV, respectively. Processed samples were thawed on ice about an hour before analysis, placed in the autosampler, and kept at 5 °C before injection. Chromatograms were acquired with Dionex Chromeleon 7 software. Analyte concentrations in tissue samples were expressed as ng/mg of frozen tissue. Data are reported as means ± SEMs. Statistical analysis used Student’s *t*-test for pairwise comparisons; a value of *p* < 0.05 was considered significant.

### 4.6. Immunohistochemistry

Mice were sacrificed with an overdose isoflurane, followed by intracardiac perfusion with PBS and ice-cold 4% (*w*/*v*) PFA in PBS (pH 7.4). The brain was dissected from the skull and post-fixed in 4% PFA for another 48 h at 4 °C. Brains were cut into 25 μm cryotome sections. Double-labeling was performed as described (Nuber et al., 2013). Briefly, sections were blocked in 10% normal donkey serum and incubated overnight at 4 °C with antibodies to hu αS (15G7, 1:500; Enzo), anti phosphorylated (pSER 129) αS (ab51253; 1:2000; Abcam), tyrosine hydroxylase (TH) (ab152, 1:500; Millipore). This was followed by incubation with FITC-conjugated secondary antibodies (1:1000 in PBS) for 4 h at RT. Nile Red was diluted 1:500.000 in staining buffer (Nile Red Kit; ab228553, abcam), applied for 15 min, washed five times for each 5 min in PBS at the final staining step, then embedded with DAPI-containing mounting medium (Vectashield). Confocal microscopy was conducted with an Axiovert 35 microscope (Zeiss) mounted on a MRC1024 laser scanning confocal microscope (Bio-Rad). An Image J plug-in called “particle analyzer” was used to analyze LD size, counts and distribution (%area). “Colocalization highlighter” created a mask of either pS129 αS overlapped with total αS and Nile Red pixels.

### 4.7. RNA

Total RNA samples were isolated from cortical brain bits using mirVana miRNA Isolation Kit (AM1561), and RNA concentrations were determined by Nanodrop. The samples were converted into cDNA using Applied Biosystems High-capacity cDNA reverse transcription kit (#4368813). Afterwards, the cDNA was mixed with Taqman Fast Universal PCR Mix (#44444553) for qPCR analysis using Taqman Gene Expression primers for LIPE (HSL) (Mm00495359_m1) and GAPDH (Mm99999915_g1).

### 4.8. Assessment of striatal DA fiber integrity

25-μm-free floating sections were rinsed in TBS (0.15 M NaCl, 0.1 M Tris-HCl, pH 7.5) and endogenous peroxidase activity quenched with 0.6% H_2_O_2_ in TBS for 30 min at RT. Unspecific protein binding was blocked with 3% normal donkey serum in TBS containing 0.3% Triton X-100. Sections were incubated with rabbit TH (AB152, 1:500, Millipore) over night at 4 °C. Following 3 rinses with TBS, sections were incubated with secondary anti-rabbit (1:1000, Dianova 711–065–152) in TBS containing 3% normal donkey serum, washed, and subsequently transferred into ABC solution (1:500 in PBS; Vectastain Elite Kit, Vector Laboratories) for 1 h and visualized with 3,3′-diaminobenzidin (DAB). Brain sections across a + 4.8 to +3.5 interaural range were chosen, referring to the Paxinos and Franklin mouse brain atlas (Paxinos and Franklin, 2001). Sections from all genotypes were simultaneously stained and digitized using constant imaging settings (Stereo-investigator, MicroBrightField, Colchester, VT), and subsequent analyses were performed on a blinded basis. Images were converted to gray scale, and the mean gray value intensity was measured in the caudate/putamen (CPu) and in the adjacent corpus callosum (cc) to correct for signal background. Mean gray values were converted to uncalibrated optical density (UOD) using ImageJ 1.46r software (NIH). The UOD of TH signal in the CPu was calculated by the formula CPu_final(UOD)_ = CPu_(UOD)_ – cc_(UOD)_, similar to a previously published study.

### 4.9. Behavioral testing

To assess motor function and coordination in walking mice, automated gait analysis was performed using Treadscan (Cleversys Inc., Reston, VA). Gait patterns of 3–6 mo mice were measured for 25 s at a speed of 13 cm/s on a transparent running belt illuminated by a LED light and reflecting footprints captured by a video camera positioned underneath the walkway.

Motor skill learning was evaluated using an accelerating rotarod (Ugo Basile), and time spent on the rod was recorded. The first two days consisted of a habituation trial at constant speed (4 rpm for 5 min), followed by two trials of 4–40 rpm progressive acceleration within 5 min. Motor coordination was evaluated by comparing the mean latency to fall between groups between these two trials.

### 4.10. Lipid sample preparation, profiling, and analysis (Lipotype)

Lipid profiling of tissue samples was performed as previously described (Fanning et al., 2022). Mass spectrometry-based lipid analysis was performed by Lipotype GmbH (Dresden, Germany) as described (Sampaio et al., 2011). Lipids were extracted using a two-step chloroform/methanol procedure (Casanovas et al., 2014) (Ejsing et al., 2009)., Samples were spiked with internal lipid standard mixture containing: cardiolipin 14:0/14:0/14:0/14:0 (CL), ceramide 18:1;2/17:0 (Cer), diacylglycerol 17:0/17:0 (DAG), hexosyl- ceramide 18:1;2/12:0 (HexCer), lyso-phosphatidate 17:0 (LPA), lyso- phosphatidylcholine 12:0 (LPC), lyso-phosphatidylethanolamine 17:1 (LPE), lyso-phosphatidylglycerol 17:1 (LPG), lyso-phosphatidylinositol 17:1 (LPI), lyso-phosphatidylserine 17:1 (LPS), phosphatidate 17:0/17:0 (PA), phosphatidylcholine 17:0/17:0 (PC), phosphatidylethanolamine 17:0/17:0 (PE), phosphatidylglycerol 17:0/17:0 (PG), phosphatidylinositol 16:0/16:0 (PI), phosphatidylserine 17:0/17:0 (PS), cholesterol ester 20:0 (CE), sphingomyelin 18:1;2/12:0;0 (SM) and triacylglycerol 17:0/17:0/17:0 (TAG). After extraction, the organic phase was transferred to an infusion plate and dried in a speed vacuum concentrator. First step dry extract was resuspended in 7.5 mM ammonium acetate in chloroform/methanol/ propanol (1:2:4,V:V:V) and second step dry extract in 33% ethanol solution of methylamine in chloroform/methanol (0.003:5:1; V:V:V). All liquid handling steps were performed using Hamilton Robotics STARlet robotic platform with the Anti Droplet Control feature for organic solvents pipetting. Samples were analyzed by direct infusion on a QExactive mass spectrometer (ThermoScientific) equipped with a TriVersa NanoMate ion source (Advion Biosciences). Samples were analyzed in both positive and negative ion modes with a resolution of Rm/z = 200 = 280,000 for MS and Rm/z = 200 = 17,500 for MSMS experiments, in a single acquisition. MSMS was triggered by an inclusion list encompassing corresponding MS mass ranges scanned in 1 Da increments. Both MS and MSMS data were combined to monitor CE, DAG, and TAG ions as ammonium adducts; PC, PC O-, as acetate adducts; and CL, PA, PE, PE O-, PG, PI, and PS as deprotonated anions. MS only was used to monitor LPA, LPE, LPE O-, LPI, and LPS as deprotonated anions; Cer, HexCer, SM, LPC, and LPC O- as acetate adducts. Data were analyzed with in-house developed lipid identification software based on LipidXplorer (Herzog et al., 2012). Data post-processing and normalization were performed using an in-house developed data management system. Only lipid identifications with a signal-to-noise ratio > 5, and a signal intensity 5-fold higher than in corresponding blank samples were considered for further data analysis.

### 4.11. Quantification and statistical analysis

Details regarding each statistical test, biological sample size (n) and *p* value can be found in the corresponding figure legends. All data are represented as mean ± SEM. SEM represents variance within a group. In all experiments, the genotypes can be found in the corresponding legends. Data were collected and processed side by side in randomized order for all experiments; most analyses were routinely performed blind to the conditions of the experiments. Unpaired, two-tailed *t*-tests were used for comparison between two groups, with *p* < 0.05 considered significant. For all comparisons involving multiple variables, a two-way ANOVA was performed followed by Tukey’s post hoc test for multiple comparison using p < 0.05 for significance. An average abundance heatmap for each cohort is also provided to highlight the more abundant FAs and data were examined by standard principal component analysis (lipotype, Dresden). For all experiments, between 3 and 6 (biochemistry, histology, lipid profiling) and 4–9 (behavior) animals per experiment were used, with the number per group stated in each figure legend. All statistical analyses were preformed using GraphPad Prism.

## Supplementary Material

1

## Data Availability

Data will be made available on request.
