# Supplementary material for: Reducing the lipase LIPE in mutant α-synuclein mice improves Parkinson-like deficits and reveals sex differences in fatty acid metabolism
Source: Neurobiol Dis. Author manuscript; Available in PMC 2024 Nov 20. (PMC11577057; doi:10.1016/j.nbd.2024.106593)
Supplement: 1 [file NIHMS2032226-supplement-1.docx]

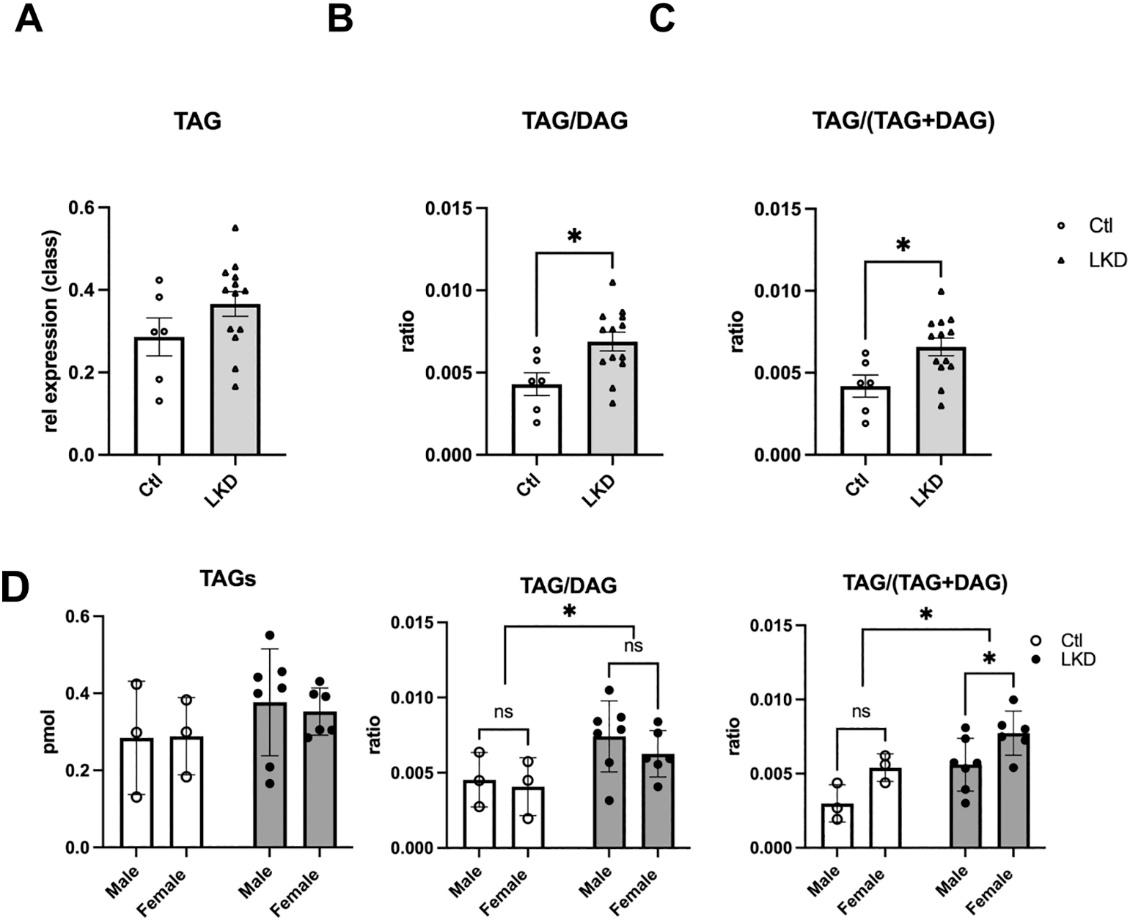


Suppl Fig. 1. Statistically significant changes in the balance of TAGs plotted against (**A**) the total of DAGs or (**B**) the sum of total TAGs and DAGs. (**C**) Sum of TAGs. (**D**) Data in A-C separated for sex. Two-tailed unpaired *t-*Test between Ctl (C57Bl6) and Lipe-Knockdown (LKD) mouse cortex or (D) 2-way ANOVA post Tukey. * *p* < 0.05.


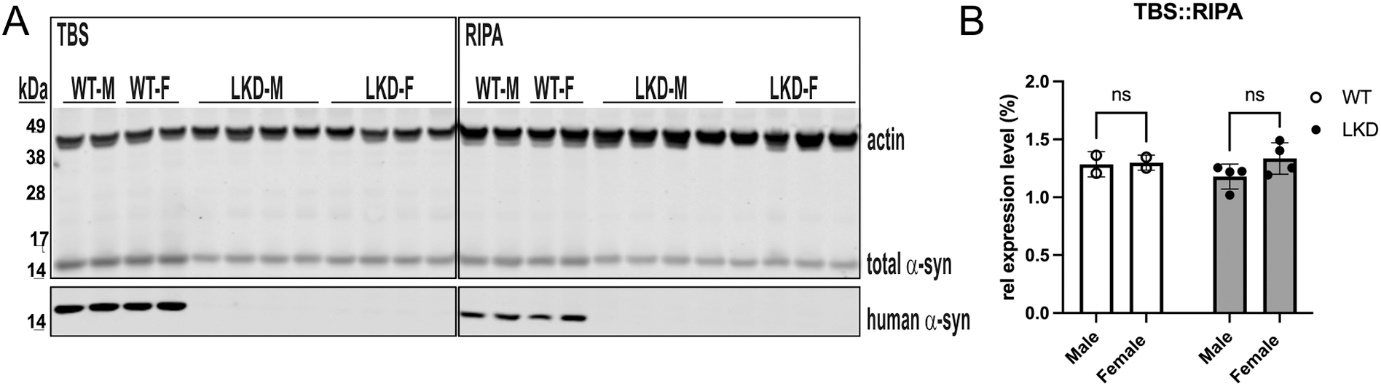


Suppl Fig. 2. No differences in solubility between male and female LKD and in relation to phenotypically normal WT aS overexpressing mice. WB (non-crosslinked) of sequentially extracted TBS-(soluble), RIPA-(insoluble) extracts of cortical brain bits and quantification (graph) of the TBS::RIPA ratio showing no differences between male and female LKD and vs. WT mice. Data are mean ± SEM. Two-way ANOVA post-hoc Tukey. n.s., non-significant.
